# Wnt6 plays a complex role in maintaining human limbal stem/progenitor cells

**DOI:** 10.1038/s41598-021-00273-y

**Published:** 2021-10-22

**Authors:** Clémence Bonnet, Denise Oh, Hua Mei, Sarah Robertson, Derek Chang, Jean-Louis Bourges, Francine Behar-Cohen, Jie J. Zheng, Sophie X. Deng

**Affiliations:** 1 Stein Eye Institute, University of California, Los Angeles, 200 Stein Plaza, Los Angeles, CA 90095 USA; 2 INSERM, UMRS1138, Team 17, From Physiopathology of Ocular Diseases to Clinical Development, Paris University, Centre de Recherche des Cordeliers, and Ophthalmology Department, Cochin Hospital, AP-HP, 75014 Paris, France; 3 Department of Ophthalmology, University of North Carolina, Chapel Hill, NC USA; 4 Molecular Biology Institute, University of California, Los Angeles, CA USA

**Keywords:** Cell growth, Cell signalling, Adult stem cells

## Abstract

The corneal epithelium is consistently regenerated by limbal stem/progenitor cells (LSCs), a very small population of adult stem cells residing in the limbus. Several Wnt ligands, including Wnt6, are preferentially expressed in the limbus. To investigate the role of Wnt6 in regulating proliferation and maintenance of human LSCs in an in vitro LSC expansion setting, we generated NIH-3T3 feeder cells to overexpress different levels of Wnt6. Characterization of LSCs cultured on Wnt6 expressing 3T3 cells showed that high level of Wnt6 increased proliferation of LSCs. Medium and high levels of Wnt6 also increased the percentage of small cells (diameter ≤ 12 µm), a feature of the stem cell population. Additionally, the percentage of cells expressing the differentiation marker K12 was significantly reduced in the presence of medium and high Wnt6 levels. Although Wnt6 is mostly known as a canonical Wnt ligand, our data showed that canonical and non-canonical Wnt signaling pathways were activated in the Wnt6-supplemented LSC cultures, a finding suggesting that interrelationships between both pathways are required for LSC regulation.

## Introduction

Corneal transparency and avascularity are vital properties to ensure a clear optical path and subsequent adequate vision. Proper renewal of the corneal epithelial cells is necessary to preserve these functions. The limbus, which is the junction between the sclera and cornea^1^, plays a major role in the maintenance and renewal of corneal epithelial cells^2–4^. A population of slow-cycling adult stem cells located in the limbus, the limbal stem/progenitor cells (LSCs), are responsible for the long-term homeostasis of the corneal epithelium^2,3^. Damage to the LSCs or their immediate microenvironment, the stem cell niche, results in limbal stem cell deficiency (LSCD). Clinical presentation of LSCD includes conjunctivalization of the ocular surface, inflammation, corneal neovascularization, loss of corneal transparency and subsequent loss of vision^5–8^.

Corneal transplantation is not an effective approach to treat LSCD, because the patient’s residual LSCs are absent/insufficient to epithelialize and maintain the health of the graft^9^. Because successful LSCD treatment requires restoration of LSCs, surgical techniques have been optimized to reduce the amount of donor tissues^10,11^ and cell-based therapies have been developed based on LSCs expansion in culture^12–14^. To further optimize the outcomes of transplantation of LSCs produced by ex vivo expansion, it is critical to identify limbal niche factors involved in LSC regulation, decipher their functions and hopefully use them as therapeutic tools.

The in vivo homeostasis of LSCs is regulated by close interactions with their microenvironment, commonly referred as the stem cell niche. The niche is a three-dimensional, stem cell-sheltering, highly organized structure, composed of extra-cellular matrix, melanocytes, limbal stromal cells, mesenchymal corneal cells, nerves, vessels, and soluble factors^7^. Regulation of proliferation and differentiation of LSCs is the result of complex molecular crosstalks with neighboring niche cells, soluble factors, and extracellular membrane components^15^. Maintaining LSCs in culture requires mimicking the in vivo niche, which must be able to provide all the necessary factors for the proliferation and survival of LSCs, and modulate the multiple signaling pathways present in the niche, including Notch^16^ and Wnt^7^.

Cultivation of human epithelial cells was initially developed by Howard Green in the 1960s^17^. This epithelial cell expansion method consisted of a co-culture of human epidermal keratocytes with 3T3-mouse embryonic fibroblasts as feeder cells, leading to the first cell-based therapy using cultured cells^18,19^. The cultivation method was later applied to LSCs by Pellegrini et al.^12^. Favorable long-term outcome has been reported^13^, which indicates the ability of the NIH-3T3 feeder cells to serve as a surrogate niche. However, the success rate has not improved over the last 2 decades. Further improvement of the culture condition by supplementing additional niche factors could improve the clinical outcome.

We previously reported that one of the critical factors of the LSC niche both in vivo^20,21^ and in vitro^22,23^, is Wnt signaling. Four Wnt ligands, Wnt2, 6, 11 and 16b had a higher expression in the limbus compared to the cornea^20,21^. Wnt ligands are small lipid-modified proteins that act over a relatively short distance and serve as niche factors^24^. Wnt signaling is required for the survival of LSCs^22^. However, characterization of the individual Wnt functions has not been conducted. Elucidation of the molecular events involved in the maintenance of the LSC is necessary for the fine manipulation of the biological factors involved in their ex vivo expansion.

Wnt6, a Wnt family member responsible for the maintenance of epithelia during vertebrate embryogenesis^25,26^, is upregulated and preferentially expressed at the basal limbal epithelial layer^20^. Frizzled 7 (Fzd7) which is present in the basal layer of limbal epithelium is a receptor of Wnt6^23,25^. Therefore, Wnt6 possibly signals via Fzd7 and may be an important player in the LSC niche. NIH-3T3 cells do not express Wnt6. Consequently, if Wnt6 does play a vital role in the maintenance of the LSC niche, NIH-3T3 feeder cells may not provide an optimal niche for LSCs. In the currently study, the role of Wnt6 in the regulation of LSCs was investigated by supplementing Wnt6 in the LSC culture via ectopic expression of Wnt6 in the NIH-3T3 feeder cells.

## Results

### Wnt6 mRNA is expressed in the human limbus and in Wnt6-3T3

**Figure 1:**
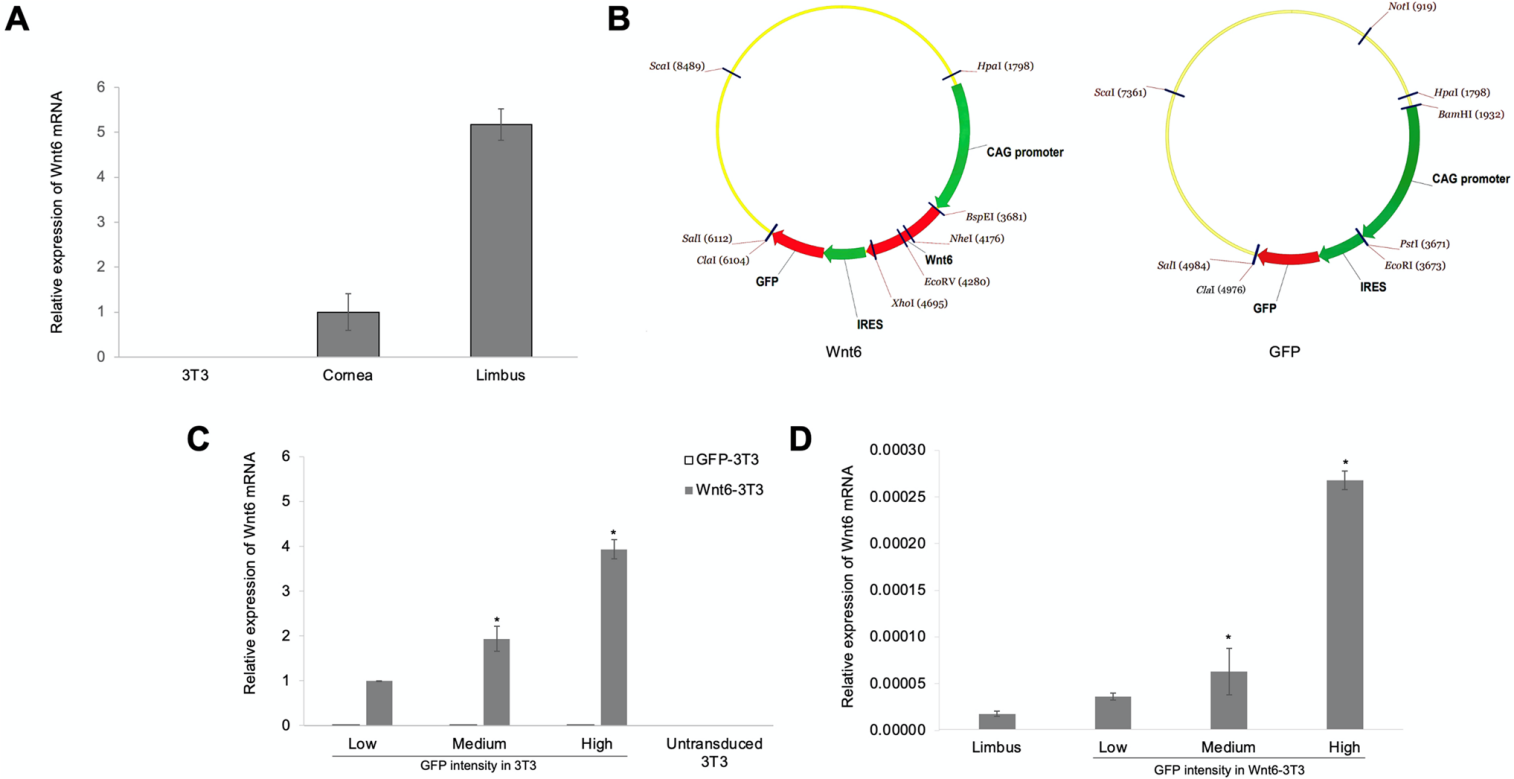
Expression of Wnt6. (**A**) mRNA from NIH-3T3 feeder cells, human corneal epithelium, and human limbal epithelium were reverse-transcribed and quantified by qRT-PCR. (**B**) NIH-3T3 feeder cells were transduced with lentiviral constructs over-expressing Wnt6 with an IRES-GFP reporter (Wnt6-3T3) or lentivirus containing the IRES-GFP reporter only (GFP-3T3) as the control. (**C**) Transduced 3T3 cells were sorted into 3 groups (low, medium, and high) based on their GFP intensities. The levels of Wnt6 expression in the 3 groups were examined by qRT-PCR. All mRNA levels were calculated as 2^−ΔCt^ values and normalized to GAPDH. *P ≤ 0.05 in comparison to the corresponding GFP-3T3 controls. Data are means ± SEM of 4 experiments. (**D**) The levels of Wnt6 mRNA expression in the low-, medium-, and high-Wnt6-3T3 cells were compared with human limbal expression of Wnt6 mRNA. All mRNA levels were calculated as $2^{{( - \Delta {\text{C}}_{{\text{t}}} )}}$ values and normalized to 18S. Data are means ± SEM of 3 experiments. *P ≤ 0.0001 in comparison with the limbus.

**Figure 2:**
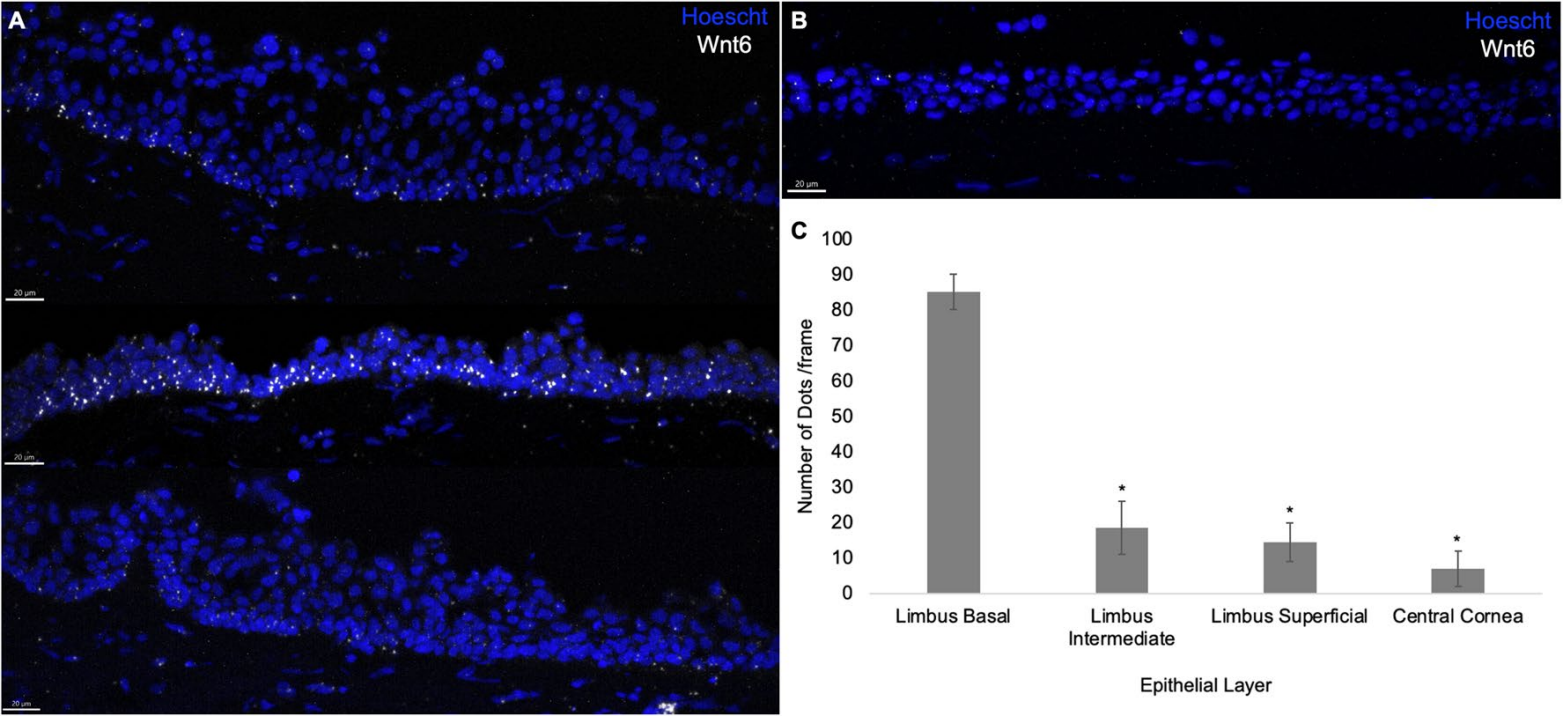
RNAscope fluorescence in situ hybridization assay. RNAscope assay showing high expression of Wnt6 mRNA in the human limbus. (**A**) Wnt6 mRNA expression in the limbus. (**B**) Wnt6 mRNA expression in the central cornea. (**C**) Wnt6 mRNA was preferentially expressed at the basal limbal epithelial layer compared with the intermediate and superficial limbal layers and with the central cornea (all *P* < 0.05 in comparison with the basal limbal epithelial layer). Data are means ± SEM of 2 independent experiments.

Quantitative (q) RT-PCR showed that the expression of Wnt6 mRNA was 5.17-fold greater in the limbus than in the central cornea (Fig. 1A). The level of endogenous Wnt6 mRNA expression in the NIH-3T3 feeder cells was minimal or absent. To supplement Wnt6 in LSC cultures, the NIH-3T3 feeder cells were transduced with a modified lentiviral vector containing the full Wnt6 coding sequence (Wnt6-3T3). The vector, pRRL.CAG.Wnt6.IRES.GFP, induced the expression of Wnt6 under a CAG promoter, and the expression of green fluorescent protein (GFP) under an internal ribosome entry site (IRES) element (Fig. 1B). Wnt6-3T3 cells were sorted on the basis of GFP fluorescence intensity (low, medium, and high). NIH-3T3 cells that expressed GFP only, at low-, medium-, and high-levels, were sorted and used as controls (GFP-3T3). Correlation between GFP intensity and Wnt6 mRNA levels were verified by qRT-PCR (Fig. 1C). In human limbus, mRNA level of Wnt6 expression was comparable to the level in low-Wnt6-3T3 cells (Fig. 1D). In situ hybridization using the RNAscope V2 Fluorescent assay (ACD, Newark, California) confirmed the importance of Wnt6 in the human limbus^20^, as Wnt6 mRNA was highly expressed, preferentially in the basal limbal epithelial cell layer (Fig. 2 and Supplemental Fig. 1).

### Effects of Wnt6 on stem cell properties of human LSCs in vitro

**Figure 3:**
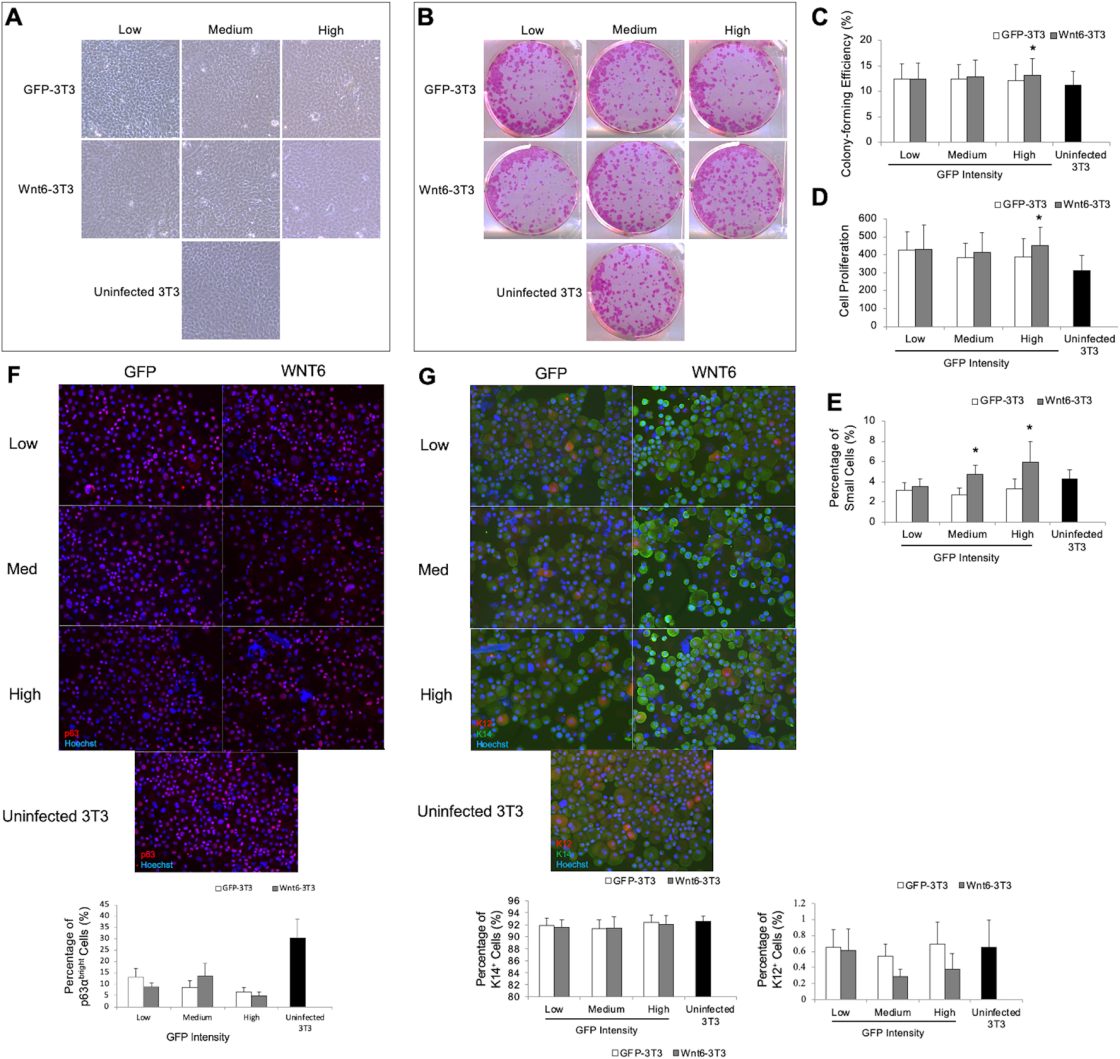
Cellular characteristics of LSCs cultured on 3T3 cells overexpressing Wnt6. LSCs were cultured on GFP-3T3, Wnt6-3T3, and uninfected 3T3 cells for 10–14 days. (**A**) Morphology of LSCs was comparable under all conditions. (**B**) CFEs of cultured LSCs were comparable. (**C**) Quantitation of CFE percentages. (**D**) Quantitation of cell proliferation. (**E**) Proportions of small cells with diameters ≤ 12 mm, (**F**) Immunostainings of p63 cells in the different groups and proportions **of** p63α^bright^ cells, (**G**) Immunostainings of K12 and K14 cells in the different groups and proportions of undifferentiated K14^+^ cells and differentiated cells determined by expression of K12. *P ≤ 0.05 in comparison with the corresponding GFP-3T3 controls. Data are means ± SEM of 4 experiments.

To determine the effects of Wnt6 on human LSCs, freshly isolated limbal epithelial cells were seeded on Wnt6-3T3 feeder cells that expressed low-, medium-, and high- levels of Wnt6 and their respective low-, medium-, and high-GFP-3T3 controls. The cell morphology was similar in the controls and Wnt6 supplemented cultures (Fig. 3A), indicating that Wnt6 expression had no immediate cytotoxic effect on LSC growth. Analysis of the colony-forming efficiency (CFE), a classic functional analysis of the clonogenic capacity of the progenitor cell population, showed that the CFE was significantly increased in high-Wnt6-3T3 cells (13.22%) as compared to the control high-GFP-3T3 cells (12.19%, 1.03% increase; *P* < 0.05) and the uninfected NIH-3T3 cells (11.26%, 1.96% increase; *P* < 0.05) (Fig. 3B–D).

Stem cells must conserve resources in times of quiescence and replication, which usually results in smaller cell size from reduced cytoplasmic/nuclear ratios^27,28^. This trend is observed in the limbus with stem/progenitor cells being about 12 µm or smaller^29,30^. Quantification of cultured cells revealed that the percentage of small cells (≤ 12 µm) significantly increased for cells cultured under medium (2.01%, *P* < 0.05) and high (2.64%, *P* < 0.05) levels of Wnt6 (Fig. 3E). We also examined expression of p63α^bright^, the currently accepted LSC marker^31^, however, expression was reduced in comparison to uninfected controls (Fig. 3F). To demonstrate that cultured LSCs maintained undifferentiated status, we analyzed populations for cytokeratin (K)14, a marker for undifferentiated cells^32^. Comparison between all treatment and control groups showed comparable percentages of cells positive for K14 (Fig. 3G). The presence and maintenance of LSCs is further demonstrated by the significant decrease detected in K12^+^ cells (Fig. 3H), the differentiation marker^33^. The expression of K12^+^ in the cultured cells was significantly reduced in both medium- (0.25%, *P* < 0.05) and high- (0.31%, *P* < 0.05) Wnt6-3T3 cultures, demonstrating the maintenance of a progenitor state. Although the expression of K12^+^ decreased by a small percentage it is large considering that the maximum percentage of differentiating cells was less than 1% of the entire cultured population. Altogether, these results demonstrate that Wnt6 supports a more a suitable growth conditions for LSC expansion, consistently with findings from our previous studies^20,22^.

### Wnt6 activation of canonical signaling in LSCs

To determine which signaling pathways are activated in LSCs upon Wnt6 exposure, freshly isolated single LSCs were cultured on control 3T3 feeder cells and treated with Wnt6-conditioned media (CM). Wnt signaling may be dosage-dependent, as demonstrated by morphogen gradients, where high concentrations may predominantly initiate the canonical pathway and low concentrations may initiate non-canonical signals, or vice versa^34–36^. The canonical pathway activation is demonstrated by increasing levels of active β-catenin^37^, whereas increases in RhoA or CamKII^38^ levels can represent activation of the non-canonical PCP and Ca^2+^ pathways, respectively.

**Figure 4:**
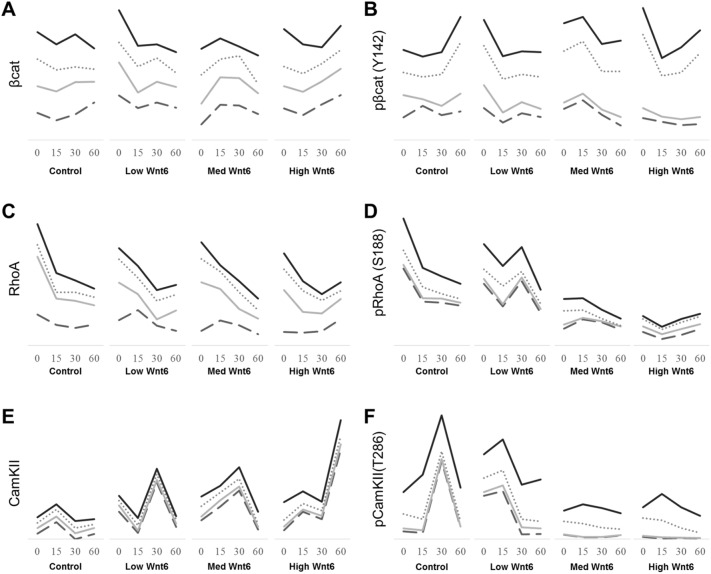
Quantitation of proteins involved in the Wnt pathway upon exposure to Wnt6. Stacked trends of individual donors measuring (**A**) β-catenin, (**B**) phosphorylated β-catenin (Y142), (**C**) RhoA, (**D**) phosphorylated RhoA (S188), (**E**) CamKII, and (**F**) phosphorylated CamKII (T286) at 0, 15, 30, and 60 min after treatment with control, low-, medium-, and high-Wnt6 conditional medium (CM). Treatment with low-Wnt6 CM resulted in immediate increases in β-catenin concentration. Treatment with medium-Wnt6 CM resulted in increases 15 min after exposure. Treatment with high-Wnt6 CM increased β-catenin 60 min after exposure. Activation of β-catenin as determined by phosphorylated β-catenin (Y142) concentrations was observed immediately after treatment with low-, medium-, and high-Wnt6 CM. Control medium also activated β-catenin at 60 min from residual 3T3-expressed Wnt ligands. RhoA expression showed a general reduction, whereas phosphorylation of RhoA was lost immediately upon treatment with medium- or high-Wnt6 CM. An increase in CamKII expression was detected almost immediately after Wnt6 treatment, and maximum levels were detected 30–60 min after Wnt6 treatment. Phosphorylation of CamKII was lost immediately during medium- or high-Wnt6 CM treatment. Each line represents the quantities from individual donors. Donor 1, solid black, 9000 cells/cm^2^; Donor 2, dotted gray, 3250 cells/cm^2^; Donor 3, solid gray, 8000 cells/cm^2^; Donor 4, gray dash, 13,500 cells/cm^2^. Normalized to GAPDH. Phosphorylated proteins were normalized to the levels of their unphosphorylated counterparts.

Analysis of β-catenin levels showed various effects, from no response to 2.3-fold increases after Wnt6 exposure. However, the trends between the donors were similar. Increases were detected immediately after treatment with low-Wnt6 CM, whereas use of med- and high-Wnt6 CM resulted in delayed β-catenin responses (Fig. 4A). We further investigated β-catenin activity, which can be measured by phosphorylation levels. Phosphorylation of β-catenin at tyrosine-142 has been found to release β-catenin from the membrane and a subsequent increase transcriptionally active β-catenin in the nucleus^39–41^. Low-, medium-, and high-Wnt6 CM increased the phosphorylation level of β-catenin by 1.75-, 2.30-, and 3.36-fold, respectively. This change in the level of phosphorylation was detected immediately upon Wnt6 exposure, confirming activation of β-catenin (Fig. 4B).

### Wnt6 regulates the non-canonical pathway

Just as β-catenin is associated with the membrane and is associated with the canonical pathway, RhoA is present at adherent junctions in cellular membranes and is associated with the non-canonical Wnt/planar cell polarity (PCP) pathway^42,43^. Figure 4C shows that the total RhoA levels generally decreased over time with all treatments of Wnt6 CM. However, analysis of phosphorylated RhoA at the serine 188 site (phospho-RhoA^S188^), which negatively regulates RhoA^38^, showed an immediate decrease in phospho-RhoA^S188^ levels upon treatment with medium- and high-Wnt6 CM by 54.18% and 76.70%, respectively (Fig. 4D). This suggests that RhoA activity was upregulated upon Wnt6 exposure.

We also examined the expression of CamKII protein, which mediates the non-canonical Wnt/Ca^2+^ pathway^44,45^. Upon Wnt6 exposure, CamKII expression increased immediately by 2.13-fold. Maximum levels were reached 30 or 60 min after treatment with Wnt6 CM in a dose dependent manner: a 3.37-fold increase was seen with low-Wnt6 CM treatment; a 3.88-fold increase, with medium-Wnt6 CM treatment; and a 5.33-fold increase, with high-Wnt6 CM treatment (Fig. 4E). Evaluation of phosphorylated CamKII levels, the activated form of the protein^45^, revealed the opposite trend in which low-Wnt6 CM treatment resulted in high phosphorylation levels, whereas medium-Wnt6 CM and high-Wnt6 CM resulted in 2.86- and 2.21-fold decreases, respectively (Fig. 4F). The differences detected immediately and 30 min after treatment suggested that divergent pathways may exist upon Wnt6 receptor binding.

## Discussion

Both Wnt6 and its receptor Fzd7 are present in the limbus^25^, suggesting a potential role in the regulation of LSCs in their niche. However, the 3T3 feeder cells used to mimic a niche environment in LSC expansion system do not express Wnt6. To explore the specific role and mechanisms of action of Wnt6 in LSCs, we developed a system in which Wnt6 was supplemented by the Wnt6-expressing 3T3 feeder layer. Low Wnt6-expressing 3T3 cells supplement Wnt6 at a similar level as in the limbal tissue, whereas high Wnt6-expressing 3T3 cells generated significantly higher level of Wnt6 in the culture. We demonstrated that medium to high levels of Wnt6 are required to increase LSC proliferation while maintaining their undifferentiated phenotype, as confirmed by the increased percentage of small cells, the stable percentage of K14^+^ cells^46^, and the decreased percentage of K12^+^ cells^33^. The percentage of p63α^bright^ cells was decreased in both the GFP-3T3 controls and Wnt6-3T3 cultures. It is possible that the GFP or the transduction protocol might affect the expression level of p63α in the LSCs, rather than affected by Wnt6 alone.

Wnt6 is expressed in the limbus in a gradient fashion as shown in Fig. 2. The basal limbal epithelial layer has the highest Wnt6 level compared to the wing and superficial layers. The critical roles of Wnt gradients for distinct intracellular responses have been reported for other cell types. For example, this gradient pattern in the limbus is similar to that reported in the intestine stem cell niche. High levels of Wnt ligands are retrieved at the bottom of the intestinal crypts, near the intestine stem cells, and function to maintain the stem cell characteristics and to promote their proliferation through Wnt/β-catenin activation^47,48^.

To further understand how Wnt6 could mechanistically promote stem/progenitor cells, we treated cultures with 3 levels of Wnt6 for short periods of time. The 3 concentrations of Wnt6 allowed us to examine the differential effect Wnt dosages can have on the downstream effectors of both canonical and non-canonical pathways, as demonstrated in other systems^34^. Interestingly, Wnt6 appears to activate both Wnt/β-catenin canonical and noncanonical pathways in LSCs. The level of the active form of phosphorylated β-catenin (Y142) increased immediately upon Wnt6 exposure at a dose dependent manner, confirming activation of Wnt/β-catenin canonical pathway in the cultivated LSCs followed by a trend of decline in the level of active phosphorylated β-catenin afterwards, whereas the opposite trend of continuous increase over time was observed in the control. Wnt6 also activates the non-canonical Wnt/Ca^2+^ at low Wnt6 level and Wnt/PCP signaling at a dose dependent manner. Given that the changes in the levels of proteins participating in the non-canonical pathways occurred as early as 15 min after the addition of Wnt6 when the β-catenin canonical pathway activation started to decline, the activation of the non-canonical pathway might be also direct. Nalesso et al. reported that low concentrations of Wnt3a in chondrocytes trigger the non-canonical Wnt/Ca^2+^ pathway and thus promote chondrocyte differentiation, whereas high concentrations of Wnt3a activate Wnt/β-catenin signaling and thus, promote proliferation of de-differentiated chondrocytes. It is possible that a single Wnt could simultaneously activate different pathways and that Wnt gradients are critical for fine stem cell regulation^49,50^. Like Wnt3a, here we demonstrated that Wnt6 can directly activate the Wnt/β-catenin canonical and noncanonical pathway effectors in LSCs. The inverse relationship of the protein levels of participants in the canonical and canonical pathways during the first hour of exposure to Wnt6 suggests that the Wnt/β-catenin canonical and noncanonical pathways activation was also time-modulated, raising the hypothesis that Wnt6 levels are finely controlled in the LSC niche, subsequently the timed activation of Wnt canonical and noncanonical pathways.

As suggested by Van Amerongen and Nusse, Wnt activation may function in integrated signaling pathways, instead of in independent ones^51^. The limbal stiffness has been shown to regulate LSCs proliferation and phenotype through interactions with several signaling pathways both in vitro and in vivo^52,53^. Gouveia et al. reported that inactivation of yes associated protein (YAP) at the basal layer, due to the softer properties of the limbus and the interactions with adhesion molecules, maintained high level of expression of ΔNp63, β-catenin, and ABCG2^53^. Subsequently, bone morphogenetic protein 4 was inhibited, preventing cell differentiation. Conversely, in the supra-basal and superficial layers, the influence of the limbal stiffness and adhesion molecules is decreased, leading to a progressive differentiation of the suprabasal cells, possibly via YAP activation. Similarly, we showed that Wnt6 is expressed in a gradient manner among the limbal layers. Overall, these results suggest that cross talks between Wnt/β-catenin signaling, and YAP-dependent mechanotransduction pathway likely work in an integrated fashion in the limbus to regulate LSC phenotype and proliferation. Moreover, crosstalk between canonical and non-canonical Wnt pathways has been reported in many cell types^49,54–56^; a growing body of literature suggests that interrelationships between these pathways are required for fine tuning of Wnt signaling^57^. To further confirm the roles of Wnt6 in the human LSC niche, exploration of downstream genes activated by the canonical and non-canonical pathways is necessary in the future studies.

The findings from our study are important in the characterization of the functions of Wnt6 in the LSC niche but also from a pharmacologic point of view. Wnt is an attractive therapeutic target for stem cell therapy, but its regulation of the multiple processes and their complexity have long slowed the task of identifying a suitable activator. Understanding how downstream biological events are individually regulated by Wnt in the LSC niche could lead to a higher degree of target specificity. Broad Wnt small molecules are able to modulate LSCs differentiation^22,58^. The availability of molecules and drugs that can selectively interfere with CamKII, RhoA, or the canonical Wnt/β-catenin pathway offers the possibility of targeting individual outcomes of Wnt stimulation, while avoiding issues related to broad Wnt activation.

In conclusion, Wnt6 is an important factor that can promote the proliferation of LSCs and maintain their phenotype in the LSC niche. Wnt6 gradients have distinct intracellular effects, modulating both canonical and non-canonical pathways; these effects suggest that modulation of Wnt6 levels could optimize cultivation of LSCs.

## Materials and methods

### Generation of NIH-3T3 cells overexpressing Wnt6

To generate NIH-3T3 feeder layers that overexpressed Wnt6 (Wnt6-3T3) and control NIH-3T3 feeder layers that overexpressed GFP only (GFP-3T3), we obtained the Wnt6 coding sequence from Dr. Eric Wexler (University of California, Los Angeles)^59^ and inserted it into a pRRL.CAG.IRES.GFP lentiviral vector (from the UCLA Integrated Molecular Technologies Core)^60,61^. The vector was packaged and transduced into NIH-3T3 cells (Howard Green, Harvard Medical School, Boston, MA, USA)^62^ through viral infection as previously described by Caviness et al.^63^. The transduced 3T3s cells were then sorted on the basis of GFP fluorescence (Eli and Edythe Broad Center of Regenerative Medicine and Stem Cell Research University of California, Los Angeles Flow Cytometry Core Resource). Low-, medium-, high-Wnt6-3T3 stable cell lines and their respective GFP-3T3 controls were selected for expansion. The similarity between the expression of GFP mRNA and Wnt6 mRNA was verified by qRT-PCR.

### Human corneoscleral tissue

Human corneoscleral tissues from healthy donors (ages, 43 to 74 years) were obtained from the San Antonio Eye Bank (San Antonio, TX) with permissions for tissue collection, Saving Sight (Kansas City, MO), and CorneaGen (Seattle, WA). All tissues were preserved in Optisol-GS (Chiron Ophthalmics, Inc., Irvine, CA) with a death-to-preservation time less than 8 h. Experiments using human tissues adhered to the tenets of the Declaration of Helsinki. The experimental protocol was exempted by the University of California Institutional Review Board (IRB#12-000363).

### Isolation and culture of human LSCs

Limbal epithelial cells (LECs), which included LSCs, were isolated as previously described^64^. Briefly, corneoscleral rims were digested with 2.4 U/mL Dispase II (Roche, Indianapolis, IN) in supplemental hormone epithelial medium (SHEM5) for 2 h at 37 °C. The limbal epithelial cell sheets were isolated and digested with 0.25% trypsin and 1 mM EDTA (Life technologies) for 5 min at 37 °C to obtain a single-cell suspension. Single LECs were seeded at a density of 200 cells/cm^2^ on subconfluent Wnt6 NIH-3T3 mouse fibroblasts that overexpressed different levels of Wnt6^62^. Growth of the NIH-3T3 cells was arrested by treatment with 4 µg/mL of mitomycin C (Sigma-Aldrich; St Louis, MO) for 2 h at 37 °C.

SHEM5 consisted of DMEM/F12 medium (Gibco, Carlsbad, CA, USA) supplemented with 5% fetal bovine serum (FBS, Life Technologies), N2 Supplement (Life Technologies), 2 ng/mL of epidermal growth factor (EGF; Life Technologies), 8.4 ng/mL of cholera toxin (Sigma-Aldrich, St. Louis, MO), 0.5 µg/mL of hydrocortisone (Sigma-Aldrich), 0.5% of dimethyl sulfoxide (DMSO; Sigma-Aldrich), penicillin/streptomycin (Life Technologies), and gentamicin/amphotericin B (Life Technologies). The medium was changed every 2–3 days, and cells were cultured for 10 to 12 days. LECs from the same donor were subjected to different culture conditions in each experiment to minimize donor variation.

### Colony-forming efficiency and cell proliferation rate

CFE was measured at the end of culture by dividing the number of colonies by the number of seeded LECs. The LSC colonies were fixed with 4% paraformaldehyde (Sigma-Aldrich) for 10 min and stained with 0.5% rhodamine B (Sigma-Aldrich) for 15 min at room temperature. The proliferation rate was calculated by dividing the number of harvested cells by the number of seeded LECs, as previously described^58^.

### Cell cultures and colony forming efficiency imaging

Images of cell cultures and CFE were taken with the digital inverted Keyence BZ-X710 fluorescence microscope (Keyence, Osaka, Japan) and the image capture system BZ-X viewer. Cell size and quantitation of expression of markers such as p63α (p63α^bright^ cells) was performed by using the BZ-X analyzer software (version 1.3.0.3) with the macro hybrid cell-count function. By specifying the mask area, the software tracked information about multiple parameters such as the intensity of fluorescence signals in different channels, cell counts, and target area measurements^65,66^. For the cell size counting, phase contrast images of single LSCs placed in a hematocytometer were used.

### Conditioned media and LSC treatment

Conditioned media (CM) was obtained from 1 × 10^4^/cm^2^ Wnt6-transduced NIH-3T3 cultured in DMEM supplemented with 10% BCS (Invitrogen) and 1% penicillin/streptomycin (Invitrogen) for 4 days. Following collection, the CM underwent centrifugation at 1000*×g* for 10 min and was concentrated by using 30-kDa Amicon Ultra Centrifugal Filters (Sigma-Aldrich). Single-cell cultures of LSCs were washed once with 1 × phosphate-buffered saline (PBS) and incubated with 200 µL of low-, medium-, and high-Wnt6-CM for specific time intervals. The LSCs were then washed twice with 1 × PBS and harvested for analysis.

### Western blot

Cells were lysed with ice cold RIPA buffer (ThermoFisher Scientific, Carlsbad, CA), and proteins in the lysate were denatured in Sample Loading Buffer (Li-cor, Lincoln, Nebraska) with 10% β-mercaptoethanol (Sigma Aldrich) for 5 min at 95 °C. Samples were loaded onto NuPAGE™ 4–12% Bis–Tris Protein Gels (ThermoFisher Scientific) and transferred onto Immobilon-FL PVDF membranes (Millipore, St. Louis, MO). Membranes were incubated for 1 h at room temperature in Odyssey blocking buffer and then overnight with the following primary antibodies: rabbit phospho-β-catenin (Abcam, ab27798; dilution ratio, 1:500); rabbit phospho-CamKII (Abcam, ab32678; dilution ratio, 1:1000), rabbit phospho-RhoA (Abcam, ab41435; dilution ratio, 1:1000); mouse active β-catenin (Millipore, 05-665; dilution ratio, 1:1000); mouse CamKII (Santa Cruz Biotechnologies, Dallas, TX, sc-5306; dilution ratio, 1:200); mouse RhoA (Abcam, ab54835; dilution ratio, 1:1000); or mouse GAPDH (Millipore, MAB374; dilution ratio, 1:500). After overnight incubation, the membranes were washed 3 times for 10 min with TBS-Tween (0.1%) (ThermoFisher Scientific). Membranes were incubated at room temperature for 1 h with the following secondary antibodies: IRDye 680LT Donkey anti-mouse IgG (Li-cor, 1:10,000) and IRDye 800CW Donkey anti-rabbit IgG (Li-cor, 1:10,000). The original output of the quantification of the fluorescent intensity from the bands of the Western blot raw images is provided in Supplemental Table 1.

### RNA extraction and qRT-PCR

Levels of Wnt6 mRNA were compared between human corneas, NIH-3T3 feeder cells, and low-, medium-, high-Wnt6-3T3 cells. Death-to-preservation time was < 6 h for all tissues used for RNA extraction. RNA extraction was performed as previously described^20,67^. Briefly, the cornea and limbal epithelia along with the immediate adjacent stroma were dissected from donor tissues and stored at − 80 °C until RNA extraction was performed. Homogenization of tissue samples was achieved by using a serrated homogenizer (Omni International, Marietta, GA). Total RNA was extracted (Qiagen RNeasy Mini Kit; Qiagen). The quantity and quality of total RNA were assessed by a spectrophotometer (NanoDrop 1000; NanoDrop, Wilmington, DE) and bioanalyzer (2100 Bioanalyzer; Agilent Technologies, Santa Clara, CA). RNA integrity number > 8 and minimal RNA degradation were required for subsequent experiments. Reverse transcription was performed by using the Superscript III Reverse Transcriptase (ThermoFisher Scientific). The relative abundance of transcripts was detected by qRT-PCR (KAPA SYBR FAST qPCR Master Mix; Stratagene, La Jolla, CA) and compared with transcript levels in NIH-3T3 cells and Wnt6-3T3 cells. GAPDH and 18S were used as internal controls to normalize the fluorescence level by using the 2^(−ΔCt)^ formula. Three donors were used to compare Wnt6 mRNA in corneal tissue with that in NIH-3T3 cells. Three additional donors were used to compare Wnt6 mRNA level in corneal tissue with that in Wnt6-3T3 cells. Reactions were performed in triplicate. The primers used for qRT-PCR are listed in Supplemental Table 2.

### Immunocytochemistry and quantitative analysis

Cultured colonies were incubated for 2 h in SHEM5 growth medium supplemented with 2.4 U/mL Dispase II (Roche) at 37 °C. Single cells were obtained with 0.25% trypsin and 1 mM EDTA (Gibco) for 7 min at 37 °C. Harvested cells underwent cytospinning onto slides by a cytocentrifuge (Cytofuge; ThermoFisher Scientific), and these slides were stored at − 80 °C until further use.

Slides were fixed with 4% paraformaldehyde at room temperature for 15 min, washed 3 times with PBS, blocked and permeabilized with 1X PBS containing 1% bovine serum albumin (BSA) and 0.5% Triton X-100 (Sigma-Aldrich) for 30 min at room temperature. Slides were incubated with primary antibodies in 1X PBS containing 1% BSA and 0.1% Triton X-100 overnight at 4 °C in a moist chamber. Primary antibodies used were the following: anti-K12 (Santa Cruz Biotechnologies, sc-25722; dilution ratio, 1:100); anti-K14 (ThermoFisher Scientific, MS-115-R7); and anti-p63α (Cell Signaling Technologies, 4892; dilution ratio, 1:100). After incubation, cells were washed with 1X PBS, incubated with the following secondary antibodies: goat anti-mouse IgG conjugated to AlexaFluor 488 (ThermoFisher Scientific, A11029, 1:500) or goat anti-rabbit IgG conjugated to AlexaFluor 546 (ThermoFisher Scientific, A11035, 1:500) at 20 °C for 1 h. Nuclei were labeled with Hoechst 33342 (Invitrogen, 33342, 4 µg/mL) at 20 °C for 15 min and mounted in Fluoromount medium (Sigma-Aldrich). Images were taken with a Keyence BZ-X710 inverted microscope (Osaka, Japan), and characteristics such as fluorescence intensity, number of cells, and size were evaluated with the BZ-X analyzer software (version 1.3.0.3) as described above.

### mRNA in situ hybridization using RNAscope assays

The RNAscope V2 Fluorescent Assay (ACD Biosystems, Newark, CA) was performed according to the ACD Biosystems protocol for fresh-frozen tissue. Corneal sections from 3 donors were hybridized with the Wnt6 mRNA probe. Assays were performed in 2 independent experiments for each donor. To confirm the mRNA integrity of the tissues, positive control probes targeting human housekeeping genes Polr2a, PPIB, and HPRT were visualized (Supplemental Fig. 1). The probes were amplified according to the manufacturer’s instructions and labeled with the fluorophore Opal 520 nm (Akoya Biosciences, FP1487001KT, 1:250), Opal 570 nm (Akoya Biosciences, FP1488001KT, 1:1500), and Opal 690 nm (Akoya Biosciences, SKU FP1497001KT, 1:1500). The Olympus FluoView FV1000 was used to visualize the fluorescence in situ hybridization signals. Quantitation of the dots among the limbal layers was performed with Imaris software and the surface and dots functions (V. 9.7.0, Imaris, Oxford Instruments, Oxon, UK).

### Statistical analysis

The Student’s t test was used to analyze data from at least 4 independent experiments in which 4 different donor tissues were used for cell culture. The mean ± standard error of the mean (SEM) are reported in each bar graph. Statistical significance was established with *P* values < 0.05.

## Supplementary Information

Supplementary Figure 1.

Supplementary Figure 2.

Supplementary Table 1.

Supplementary Table 2.

## Data Availability

All data and materials are available upon reasonable request.
