# Supplementary material for: Wnt6 plays a complex role in maintaining human limbal stem/progenitor cells
Source: Sci Rep. 2021 Oct 22;11:20948. doi: 10.1038/s41598-021-00273-y (PMC8536737; doi:10.1038/s41598-021-00273-y)

**Supplemental Figure 1. Image analysis of the RNAscope V2 Fluorescent Assay in human limbus and central cornea.**

A: Positive controls: Polr2a, PPIB, HPRT.

B: Negative control: DapB scrambled in all channels

C: Segmentation of basal, intermediate, and superficial layers by Imaris (surfaces tracing tool, V. 9.7.0).


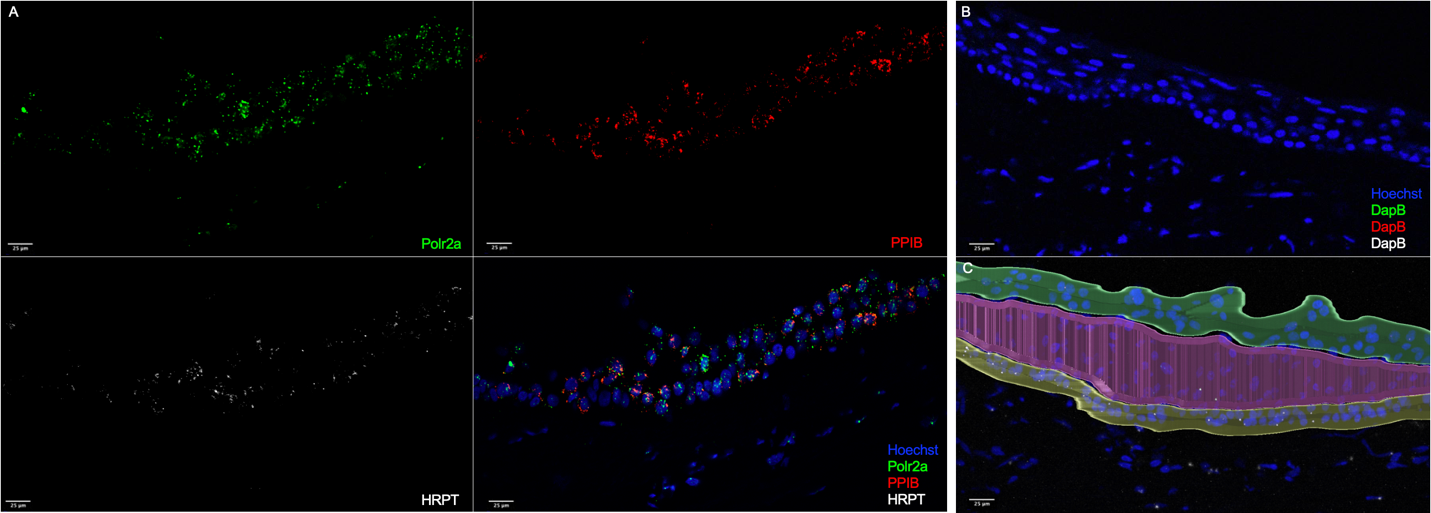

Supplement: Supplementary file 1 — Supplementary Figure 1. [file 41598_2021_273_MOESM1_ESM.docx]
