# Supplementary material for: Wnt6 plays a complex role in maintaining human limbal stem/progenitor cells
Source: Sci Rep. 2021 Oct 22;11:20948. doi: 10.1038/s41598-021-00273-y (PMC8536737; doi:10.1038/s41598-021-00273-y)
Supplement: Supplementary file 2 — Supplementary Figure 2. [file 41598_2021_273_MOESM2_ESM.docx]

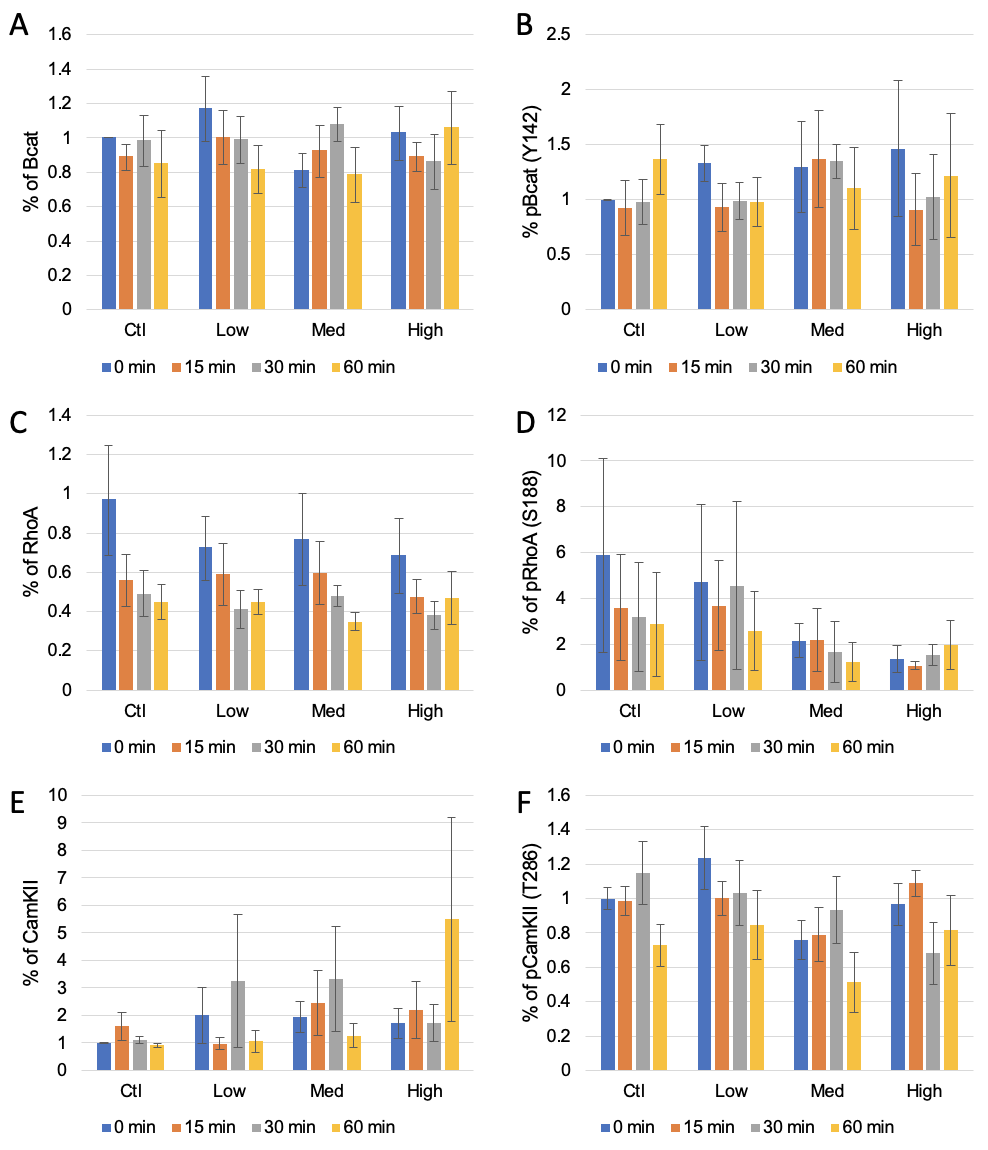


**Supplemental Figure 2.** **Quantitation of proteins involved in the Wnt pathway upon exposure to Wnt6.** Percentage of protein expression normalized to GAPDH **(A)** β-catenin, **(B)** phosphorylated β-catenin (Y142), **(C)** RhoA, **(D)** phosphorylated RhoA (S188), **(E)** CamKII, and **(F)** phosphorylated CamKII (T286) at 0, 15, 30, and 60 minutes after treatment with control (Ctl), low-, medium-, and high-Wnt6 conditional medium (CM).
