# Supplementary material for: Wnt6 plays a complex role in maintaining human limbal stem/progenitor cells
Source: Sci Rep. 2021 Oct 22;11:20948. doi: 10.1038/s41598-021-00273-y (PMC8536737; doi:10.1038/s41598-021-00273-y)
Supplement: Supplementary file 3 — Supplementary Table 1. [file 41598_2021_273_MOESM3_ESM.docx]

**Supplemental Table 1.** Original output of the quantification of the fluorescent intensity from the bands of the Western blot raw images

| **Bcat** | **Donor 1** | **Donor 2** | **Donor 3** | **Donor 4** | **Cam-KII** | **Donor 1** | **Donor 2** | **Donor 3** | **Donor 4** | **RhoA** | **Donor 1** | **Donor 2** | **Donor 3** | **Donor 4** |
| --- | --- | --- | --- | --- | --- | --- | --- | --- | --- | --- | --- | --- | --- | --- |
| CTL 0 | 1966.419 | 7062.655 | 8101.202 | 5702.758 | CTL 0 | 13.938 | 251.269 | 145.339 | 24.120 | CTL 0 | 92.461 | 171.341 | 87.987 | 34.649 |
| CTL 15 | 1273.469 | 7869.066 | 6486.761 | 5398.089 | CTL 15 | 43.301 | 259.001 | 187.999 | 23.829 | CTL 15 | 60.799 | 78.683 | 66.299 | 18.105 |
| CTL 30 | 1821.503 | 8689.535 | 4642.305 | 6901.241 | CTL 30 |  | 269.310 | 135.623 | 31.863 | CTL 30 | 52.123 | 80.138 | 38.083 | 25.637 |
| CTL 60 | 2565.309 | 5562.878 | 3791.491 | 4338.197 | CTL 60 | 12.928 | 268.526 | 101.385 | 23.533 | CTL 60 | 61.981 | 57.270 | 35.183 | 24.968 |
|  |  |  |  |  |  |  |  |  |  |  |  |  |  |  |
| LOW 0 | 2944.002 | 8631.711 | 6077.124 | 5995.609 | LOW 0 | 70.437 | 294.770 | 125.502 | 22.475 | LOW 0 | 75.569 | 111.176 | 69.130 | 32.625 |
| LOW 15 | 2434.491 | 4175.633 | 7780.205 | 7330.551 | LOW 15 | 15.835 | 99.520 | 144.978 | 33.354 | LOW 15 | 105.549 | 47.320 | 47.653 | 35.368 |
| LOW 30 | 2620.740 | 5622.699 | 7037.442 | 5298.832 | LOW 30 | 146.243 | 115.072 | 146.946 | 25.585 | LOW 30 | 58.845 | 18.634 | 55.053 | 31.808 |
| LOW 60 | 2338.184 | 5653.759 | 4197.074 | 4397.366 | LOW 60 | 31.606 | 150.210 | 110.939 | 14.359 | LOW 60 | 43.659 | 60.217 | 47.323 | 28.471 |
|  |  |  |  |  |  |  |  |  |  |  |  |  |  |  |
| MED 0 | 1053.599 | 5600.792 | 8690.571 | 4677.399 | MED 0 | 48.106 | 183.509 | 280.360 | 41.644 | MED 0 | 43.194 | 144.411 | 69.564 | 49.133 |
| MED 15 | 2147.420 | 7481.263 | 5378.419 | 3964.731 | MED 15 | 83.610 | 295.984 | 213.517 | 29.508 | MED 15 | 74.943 | 93.455 | 51.685 | 18.246 |
| MED 30 | 2448.621 | 7533.146 | 6744.658 | 6757.272 | MED 30 | 125.011 | 186.101 | 212.966 | 51.135 | MED 30 | 59.806 | 48.720 | 49.145 | 33.520 |
| MED 60 | 2031.041 | 5647.523 | 2780.694 | 6040.611 | MED 60 | 24.150 | 129.547 | 77.069 | 54.503 | MED 60 | 33.643 | 46.219 | 24.859 | 34.881 |
|  |  |  |  |  |  |  |  |  |  |  |  |  |  |  |
| HIGH 0 | 2314.619 | 6045.969 | 5790.053 | 7963.252 | HIGH 0 | 22.277 | 181.035 | 180.441 | 79.248 | HIGH 0 | 40.594 | 123.584 | 62.412 | 46.582 |
| HIGH 15 | 1890.244 | 6330.088 | 5523.312 | 6190.886 | HIGH 15 | 70.811 | 100.525 | 154.131 | 55.712 | HIGH 15 | 38.439 | 60.889 | 63.196 | 27.949 |
| HIGH 30 | 2780.809 | 6288.368 | 5292.202 | 3495.752 | HIGH 30 | 50.826 | 168.530 | 140.724 | 39.454 | HIGH 30 | 42.860 | 52.707 | 38.544 | 18.627 |
| HIGH 60 | 3486.849 | 7076.242 | 5703.670 | 5114.074 | HIGH 60 | 230.997 | 242.051 | 237.197 | 68.070 | HIGH 60 | 78.976 | 59.314 | 24.013 | 25.713 |
|  |  |  |  |  |  |  |  |  |  |  |  |  |  |  |
| **p-Bcat** | **Donor 1** | **Donor 2** | **Donor 3** | **Donor 4** | **p-CamKII** | **Donor 1** | **Donor 2** | **Donor 3** | **Donor 4** | **p-RhoA** | **Donor 1** | **Donor 2** | **Donor 3** |  |
| CTL 0 | 426.979 | 389.534 | 663.266 | 236.561 | CTL 0 | 56.742 | 471.771 | 1234.109 | 314.090 | CTL 0 | 1429.028 | 69.615 | 267.701 |  |
| CTL 15 | 476.344 | 121.941 | 522.614 | 201.061 | CTL 15 | 7.119 | 387.971 | 1232.114 | 627.945 | CTL 15 | 818.811 | 60.277 | 202.000 |  |
| CTL 30 | 446.856 | 195.839 | 528.231 | 286.360 | CTL 30 |  | 364.245 | 618.417 | 680.487 | CTL 30 | 796.247 | 70.937 | 93.711 |  |
| CTL 60 | 750.306 | 240.399 | 706.336 | 205.180 | CTL 60 | 46.712 | 160.115 | 320.528 | 467.076 | CTL 60 | 739.177 | 56.666 | 65.449 |  |
|  |  |  |  |  |  |  |  |  |  |  |  |  |  |  |
| LOW 0 | 996.340 | 487.024 | 874.190 | 285.464 | LOW 0 | 98.333 | 637.950 | 1034.976 | 319.594 | LOW 0 | 1146.181 | 85.834 | 179.307 |  |
| LOW 15 | 395.738 | 98.757 | 943.733 | 310.508 | LOW 15 | 16.697 | 340.938 | 1301.732 | 607.674 | LOW 15 | 723.762 | 46.766 | 333.382 |  |
| LOW 30 | 701.692 | 149.669 | 712.630 | 227.312 | LOW 30 | 48.873 | 483.852 | 703.047 | 524.466 | LOW 30 | 1192.214 | 70.450 | 107.019 |  |
| LOW 60 | 519.473 | 108.989 | 487.042 | 203.170 | LOW 60 | 46.062 | 499.918 | 464.803 | 356.491 | LOW 60 | 602.917 | 75.014 | 98.617 |  |
|  |  |  |  |  |  |  |  |  |  |  |  |  |  |  |
| MED 0 | 344.089 | 84.779 | 1637.044 | 240.070 | MED 0 | 21.162 | 40.756 | 2137.553 | 265.658 | MED 0 | 316.357 | 70.741 | 259.551 |  |
| MED 15 | 986.792 | 116.377 | 1026.857 | 180.533 | MED 15 | 28.623 | 97.215 | 1699.124 | 336.969 | MED 15 | 483.859 | 32.196 | 139.930 |  |
| MED 30 | 617.287 |  | 938.558 | 345.679 | MED 30 | 44.991 | 36.468 | 1155.195 | 602.238 | MED 30 | 428.325 | 21.379 | 49.096 |  |
| MED 60 | 261.422 | 115.262 | 461.720 | 349.899 | MED 60 | 7.005 | 37.195 | 277.102 | 510.311 | MED 60 | 292.233 | 60.797 | 19.372 |  |
|  |  |  |  |  |  |  |  |  |  |  |  |  |  |  |
| HIGH 0 | 483.665 | 145.800 | 1545.436 | 394.854 | HIGH 0 | 26.546 | 128.477 | 1912.209 | 494.529 | HIGH 0 | 252.862 | 103.164 | 56.279 |  |
| HIGH 15 | 320.056 | 77.980 | 816.547 | 202.285 | HIGH 15 | 8.752 | 75.788 | 1559.983 | 872.402 | HIGH 15 | 123.421 | 88.930 | 48.516 |  |
| HIGH 30 | 368.509 | 92.146 | 894.171 | 163.574 | HIGH 30 | 21.463 | 67.244 | 802.704 | 487.237 | HIGH 30 | 200.646 | 108.298 | 59.082 |  |
| HIGH 60 | 509.850 | 114.105 | 1333.495 | 211.991 | HIGH 60 | 20.058 | 57.476 | 729.400 | 689.089 | HIGH 60 | 306.046 | 89.157 | 48.640 |  |

Bcat= 𝛃catenin ; CTRL= control ; MED= medium; p-𝛃cat : Phosphorylated β-catenin (Y142).
