# Supplementary material for: Wnt6 plays a complex role in maintaining human limbal stem/progenitor cells
Source: Sci Rep. 2021 Oct 22;11:20948. doi: 10.1038/s41598-021-00273-y (PMC8536737; doi:10.1038/s41598-021-00273-y)
Supplement: Supplementary file 4 — Supplementary Table 2. [file 41598_2021_273_MOESM4_ESM.docx]

| Gene | Direction | Primer Sequence |
| --- | --- | --- |
| GAPDH | Forward | GTCTCCTCTGACTTCAACAGCG |
|  | Reverse | ACCACCCTGTTGCTGTAGCCAA |
| 18S | Forward | GTAACCCGTTGAACCCCATT |
|  | Reverse | CCATCCAATCGGTAGTAGCG |
| 𝛥NP63 | Forward | TCCATGGATGATCTGGCAAGT |
|  | Reverse | GCCCTTCCAGATCGCATGT |
| FZD7 | Forward | CGGGACCCCGGCGCGGCCGCTCCGC |
|  | Reverse | TCTCGCCCGC ACCGTGCACC |
| K12 | Forward | CCAGGTGAGGTCAGCGTAGAA |
|  | Reverse | CCTCCAGGTTGCTGATGAGC |
| K14 | Forward | GACCATTGAGGACCTGAGGA |
|  | Reverse | ATTGATGTCGGCTTCCACAC |
| WNT6 human | Forward | GAGAGTGCCAGTTCCAGTTC |
|  | Reverse | AACTGGAACTGGCACTCTCG |
| WNT6 mouse | Forward | CAGGACATCCGAGAGACAGC |
|  | Reverse | CCCATGGAACAGGCTTGAGT |

**Supplemental Table 2.** Primers used for the reverse transcriptase quantitative polymerase chain reaction
